# Effect of iron and magnesium addition on population dynamics and high value product of microalgae grown in anaerobic liquid digestate

**DOI:** 10.1038/s41598-020-60622-1

**Published:** 2020-02-26

**Authors:** Hande Ermis, Unzile Guven-Gulhan, Tunahan Cakir, Mahmut Altinbas

**Affiliations:** 1 Department of Environmental Engineering, Istanbul Technical University, 34469 Maslak Istanbul, Turkey; 2 PHI Tech Bioinformatics R&D Inc., 41400 Gebze, Kocaeli Turkey; 3 Department of Bioengineering, Gebze Technical University, 41400 Gebze, Kocaeli Turkey

**Keywords:** Biotechnology, Environmental sciences, Engineering

## Abstract

In this study, FeSO_4_ supplementation ranging from 0 to 4.5 mM, and MgSO_4_ supplementation ranging from 0 to 5.1 mM were investigated to observe the effect on the population dynamics, biochemical composition and fatty acid content of mixed microalgae grown in Anaerobic Liquid Digestate (ALD). Overall, 3.1 mM FeSO_4_ addition into ALD increased the total protein content 60% and led to highest biomass (1.56 g L^−1^) and chlorophyll-a amount (18.7 mg L^−1^) produced. Meanwhile, 0.4 mM MgSO_4_ addition increased the total carotenoid amount 2.2 folds and slightly increased the biomass amount. According to the microbial community analysis, *Diphylleia rotans*, *Synechocystis PCC-6803* and *Chlorella sorokiniana* were identified as mostly detected species after confirmation with 4 different markers. The abundance of *Chlorella sorokiniana* and *Synechocystis PCC-6803* increased almost 2 folds both in iron and magnesium addition. On the other hand, the dominancy of *Diphylleia rotans* was not affected by iron addition while drastically decreased (95%) with magnesium addition. This study helps to understand how the dynamics of symbiotic life changes if macro elements are added to the ALD and reveal that microalgae can adapt to adverse environmental conditions by fostering the diversity with a positive effect on high value product.

## Introduction

Microalgae, having no need for arable land, is an attractive source of high value products by their rapid growth. However, the main obstacle to the commercialization of algae-derived products is the high cost of production^1^. To overcome this high capital investments and operation costs, high-value co-products such as pigments, proteins, lipids, and carbohydrates should be produced to improve the economics of microalgae applications^2^ along with wastewater treatment, which is a source to obtain nutrients at a low cost. Anaerobic digestion is biodegradation of nutrient rich biomass, which is commonly used for organic matter stabilization and biogas production. Unfortunately, this process leads to produce Anaerobic Liquid Digestate (ALD)^3^, which is extremely high in ammonia and orthophosphate. Even though direct land application is considered as the most cost-effective solution due to high soil remediation properties in agriculture and reducing the cost of the logistics^4^, characteristics of the digestion effluent can cause phytotoxic effects in plants and/or contaminate the groundwater^5^. In this aspect, microalgae can be efficiently grown in liquid digestate and stabilize the effluent without any further treatment.

The effect of macro elements such as nitrogen and phosphorus on microalgae and its biochemical composition has been the focus of research. However, other macro elements such as iron and magnesium play also a critical role in a variety of metabolic pathways important for microalgae. For instance, iron (Fe) is a crucial micronutrient for almost all living organisms because of its role in metabolic processes such as DNA synthesis, respiration, and photosynthesis. It works as a cofactor for enzymes due to its ability to gain and lose electrons^6^. Magnesium (Mg), on the other hand, occupies a strategic position as the central element of the chlorophyll molecule, and all microalgal species have an absolute need for this element^7^. Although, the deficiency of iron and magnesium on microalgal growth and photosynthetic efficiency has been investigated, only a few studies focus on the influence of iron and magnesium supply on the biochemical composition. They were all in synthetic media, and only for a limited number of species.

Tap water is mostly used for diluting the wastewater, and certain type of bacteria appeared in tap water had been examined for their effects on microalgae growth^8^. Richmond and Becker^9^ demonstrated that short-term changes in growth conditions can reduce the number of those undesired microorganisms, which is important for non-axenic cultures grown in unsterilized wastewater. Undefined mixed algal culture isolated from nature is a black box that is needed to be enlightened since each undefined culture is unique and specific to its environment. Non-axenic mix microalgae consortia can perform better than unicellular culture with regard to dominancy change due to stress conditions, which prevents the culture loss and lowers the risk of system contamination. However, the mixed consortia should be observed cautiously to assure that microalgae concentration in the consortia remains higher than the bacteria culture to prevent the disappearance of algal cells^10^.

Large-scale taxonomic identification has been a challenge for mixed culture composition analysis. Metabarcoding is a novel terminology^11^ that has been used for the large-scale taxonomic identification of complex environmental samples^12–14^. DNA metabarcoding has been argued to be the next generation tool for detecting mixed species biodiversity in ecological studies and aquatic ecosystems^15,16^. Multi-marker metabarcoding, the use of multiple marker regions, is preferred to characterize mixed cultures that include prokaryotes and eukaryotes^13,17^. A wide range of prokaryotic and eukaryotic organisms can be identified via 16S rRNA, 18S rRNA and 23S rRNA barcoding analysis. More specific markers are also available such as tufA region, which was found to be an effective marker for the identification of prokaryotic (the cyanobacteria) and eukaryotic algae^12^.

The aim of this study was to elucidate how population dynamics changed, and which species were favored by Fe and Mg supplementation. The effect of these elements on biochemical composition of mixed culture and nutrient removal efficiency was also investigated. This study is the first attempt to analyze undefined algal microbiome grown in anaerobic digestate, and it will help to understand how the dynamics of symbiotic life changes if macro elements are added to the ALD. A multi-marker metabarcoding approach was used for the characterization of microorganisms in the mixed cultures in anaerobic liquid digestate by analyzing 16S rRNA, 18S rRNA, 23S chloroplast RNA and tufA marker regions. To the best of the authors knowledge, there is no other study that uses multi-marker metabarcoding approach and reports confirmation of results with 4 markers simultaneously for undefined mixed culture of anaerobic digestion, which is important for revealing the molecular diversity in detail. The results of this study will contribute to the efforts to combine digestate treatment with microalgae cultivation for an effective conversion of high strength dark wastewater into high value byproducts.

## Results and Discussion

### Effect of iron on cell growth and biochemical composition

The microalgal growth was monitored by measuring the Chlorophyll-a (Chl-a; mg L^−1^) and Suspended Solid (SS) concentrations during the experiment due to the dark color of wastewater^5^. Many studies also encountered the same problem where recent studies by Marazzi *et al*.^18^ and Huy *et al*.^19^ reported that measuring OD values for liquid digestate could be challenging due to their strong color.

**Figure 1:**
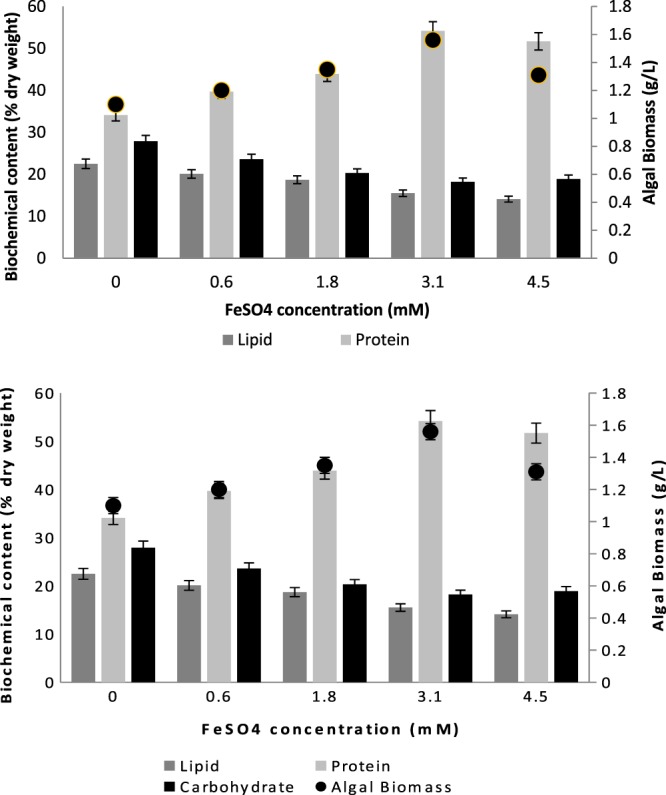
Change in the concentration of (**A**) Chl-a, (**B**) Car as a function of time for different concentrations of iron.

Iron is an essential nutrient for the survival of all organisms. It is involved in chlorophyll biosynthesis, and it enhances biomass production. Therefore, iron deficiency invariably leads to a simultaneous loss of chlorophyll and degeneration of chlorophyll structure. In order to protect the light-harvesting pigment content, including carotenoid, chlorophyll levels keep decreasing, where iron limited microalgae was reported to have lower pigment concentrations^20^. In this study, the increase of iron concentration increased both *chl-a* and *car* amount until 3.1 mM FeSO_4_ which supported the argument mentioned above. As depicted in Fig. 1, for all iron concentrations, there were 7 days of slower growth for the mixed culture to adapt to the iron supplementation. On the other hand, the control batch had rapid growth since the mixed culture was adapted to ALD for almost 3 years.

After 7 days, rapid increase of *chl-*a was observed for all batches, and the batch with 3.1 mM FeSO_4_ reached maximum *chl*-a amount (18.7 mg L^−1^) after 16 days. For the carotenoid amount, also maximum amount was observed (5.8 mg L^−1^) at 3.1 mM FeSO_4_ concentration. These parameters indicated that the efficiency of photosynthesis was related to iron nutrition as Rueter *et al*.^21^ mentioned in their study. They explained that iron was also essential for cytochromes, ferredoxin and iron sulfur proteins and absence of it not only reduced the amount of these essential components of photosynthesis but also disrupted their optimal ultrastructural arrangement, which interfered with the function of the photosynthetic apparatus. However, 4.5 mM FeSO_4_ concentration did not lead to any further increase of neither chl-a nor car concentrations (Fig. 1). This can be due to the inhibition by iron-replete where excessive levels of essential metals, like iron, can be detrimental to the organisms^22^.

**Figure 2:**
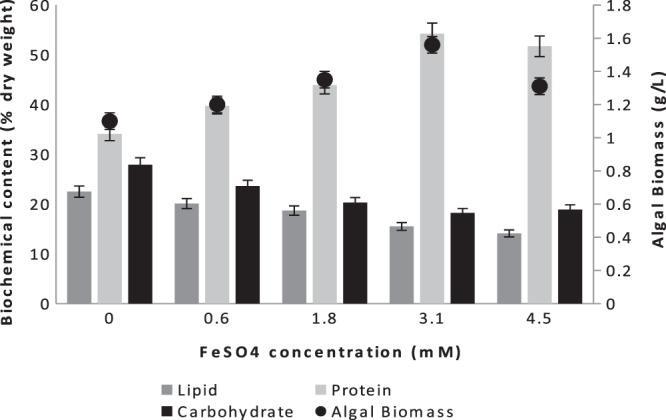
Biochemical composition and biomass amount of the mixed culture at different FeSO_4_ concentrations.

Compared with iron limitation studies, only a few experiments have been done under the iron replete conditions, and they were only with pure marine cultures grown in synthetic media. Sasireka and Muthuvelayudham^23^ applied various concentrations of FeSO_4_ ranging from 10 µM to 50 µM on *Skeletonema costatum* and concluded that 30 µM FeSO_4_.7H_2_O resulted in the highest growth rate, which was a very low concentration of ferrous source compared to our study. Moreover, Huang *et al*.^24^ studied the highest final cell densities of three different algae *(Tetraselmis subcordiformis*, *Nannochloropsis oculata* and *Pavlova viridis*) in different concentrations (0.012, 0.12, 1.2 and 12 mM) of ferric ion, and by increasing the ferric ion concentrations from 1.2 to 12 mM, specific growth rates of three microalgae decreased significantly. In their study, the microalgal cultures treated with 0.12 mM iron showed the highest final cell densities. In our study, increasing ferrous concentration from 0 to 3.1 mM enhanced the growth of mixed culture; however, at 4.5 mM, the algal growth ceased. The algal biomass reached 1.06, 1.24, 1.35, 1.56 and 1.31 mg L^−1^ when the ferrous concentrations were 0.6, 1.8, 3.1, and 4.5 mM, respectively (Fig. 2). Moreover, nutrient removal was correlated with the algal biomass productivity, whereas the highest nutrient removal for NH_3_-N and PO_4_-P was observed at 3.1 mM FeSO_4_, with 89% and 76% removal efficiency, respectively.

The growth and biochemical content of microalgae is important as a sustainable biological resource. The synthesis and accumulation of energy storage compounds can be enhanced by using appropriate variations in cultivation conditions. In this study, protein content increased from 34.1% to 54.2% when concentration of iron was increased at 3.1 mM FeSO_4_ (Fig. 2). In this study, along with an increase of protein content, the lipid and carbohydrate contents decreased simultaneously. The reason of concomitant decrease in the levels of both carbohydrates and lipids is that their synthesis pathways are related, where the energy is first stored in the form of carbohydrates and then the excess is converted into lipids^25^. The synthesis of triacylglyceride (TAG) and carbohydrate tends to compete with each other, and which one among those two types of energy storage is produced appears species specific^26^. Liu *et al*.^27^ reported that *Chlorella vulgaris* supplemented with 10 µM iron exhibited a lipid content up to 56.6% of the biomass by dry weight, which was 3–7 fold higher than lower iron concentrations. Moreover, in Ghafari *et al*.^28^’ s study, *B*. *sudeticus*, *C*. *sorokiniana*, *C*. *vulgaris*, and *E*. *oleoabundans* showed 10, 60, 18, and 36% increase in lipid content at 4.32 µM Fe presence, respectively. This was expected as Fe increases overall neutral lipid accumulation due to the down-regulation of iron requiring fatty acid desaturase enzymes. In this study, lipid content decreased since dominancy in the culture changed in favor of a low-lipid-yield microalgae species. Overall, 3.1 mM ferrous addition into ALD increased the total protein content 60% along with highest biomass (1.56 g L^−1^) and highest chlorophyll-a amount (18. mg L^−1^). Therefore, 3.1 mM FeSO_4_ addition was found to be the optimum iron concentration for a positive effect on algal biomass for further applications. However, in this study, mixed algal culture was analyzed rather than pure algal culture; therefore, the dominancy changes also affected the total biochemical composition of the system.

After the biochemical analyses of the mixed culture, the fatty acid compositions were determined. The composition of fatty acids of mixed culture was mainly Palmitic acid (C16:0) and Stearic acid (C18:0) for saturated fatty acids (SFU); Pentadecenoic acid (cis-10) (15:1), Elaidic acid (18:19t), Oleic acid (18:19c) and Gondoic acid 20:1 for monousaturated fatty acids (MUFA); and Linolelaidic acid (18:2 9t), Linoleic acid (18:2 9c), α-Linolenic acid (18:3) and Eicosadienic acid (20:3) for polyunsaturated fatty acids (PUFA); which were the most common fatty acid profiles of algae. The highest amount of Palmitic acid (C16:0) and Stearic acid (C18:0) were observed at 3.1 mM FeSO_4_ concentration with 46.6% and 8.3%, respectively. As 4.5 mM FeSO_4_ and control cultivation showed similar results for growth; the fatty acid composition concentrations had also similarities. Both of them had C18:1 n-9c (oleic acid), whereas other concentrations did not have C18:1 n-9c which is the most representative fatty acid of MUFA mainly containing in olive oil. Moreover, C20:3 disappeared at the iron concentrations of 3.1 and 4.5 mM, which indicates that different iron concentrations could affect the composition of the fatty acids and can be effectually altered by changing iron concentrations. Since the unsaturation grade affects the cold flow, stability and ignition quality of diesel fuel^26^, C18:3 amount was limited as <12% (w/w) by European biodiesel standard EN14214^29^. According to the results from this study, C18:3 amount was measured between 1.3–3.1%, which examined the high quality of the biodiesel (Table 1).

**Table 1:** FAME profiles for mixed microalgae under different iron concentrations.

Fatty Acids	FeSO_4_ Concentration (mg L^−1^)				
	(Mean ± SD^*^), n = 3				
	0^a^	130^b^	400^c^	700^d^	1000^e^
	Fatty Acid Composition (%, w/w)				
***Saturated fatty acids (SFU)***					
16:0	*32*.*4* ± *2*.*9*	**39**.**5** ± **2**.**2**	**44**.**3** ± **3**.**2**	**46**.**6** ± **5**.**5**	**41**.**6** ± **3**.**4**
18:0	*4*.*7* ± *0*.*22*	**5**.**6** ± **0**.**25**	**6**.**7** ± **0**.**21**	**8**.**3** ± **0**.**1**	**6**.**1** ± **0**.**08**
***Monounsaturated fatty acids (MUFA)***					
15:1	*6*.*6* ± *0*.*34*	**12**.**6** ± **1**.**56**	**8**.**8** ± **0**.**99**	**10**.**5** ± **0**.**5**	**13**.**4** ± **0**.**27**
18:1 n-9t	*10*.*1* ± *1*.*5*	**21**.**6** ± **3**.**4**	15.7 ± 3.4	**23**.**1** ± **0**.**34**	—
18:1 n-9c	*7*.*6* ± *0*.*44*	—	—	—	**9**.**2** ± **0**.**05**
20:1	—	—	**1**.**8** ± **0**.**5**	—	—
***Polyunsaturated fatty acids (PUFA)***					
18:2 9t	*4*.*1* ± *0*.*11*	4.6 ± 0.33	—	4.1 ± 0.09	**3**.**8** ± **0**.**11**
18:2 9c	*6*.*8* ± *0*.*23*	**11**.**5** ± **0**.**95**	**10**.**2** ± **0**.**1**	6.4 ± 0.35	**16**.**6** ± **036**
18:3	*1*.*3* ± *0*.*09*	**2**.**1** ± **0**.**06**	**3**.**1** ± **0**.**12**	**2**.**8** ± **0**.**05**	**1**.**5** ± **0**.**04**
20:3	*0*.*26* ± *0*.*01*	**2**.**4** ± **0**.**08**	**6**.**4** ± **0**.**45**	—	—

^*^SD: Standard Deviation.

Bold font indicates statistically significant difference with respect to 0 mg/L Fe concentration (t-test, P < 0.05).

### Effect of magnesium on cell growth and biochemical composition

Since magnesium is the center element of chlorophyll, a higher chlorophyll-a amount was expected with increasing MgSO_4_ concentrations; however, there was no significant differences on neither biomass amount nor chlorophyll amount (Table 2). However, the highest carotenoid amount between all batches and concentrations was observed at 0.4 mM MgSO_4_ concentration (7.4 mg L^−1^), where it was 3.3 mg L^−1^ at control cultivation and 5.8 mgL^−1^ at 5.1 mM MgSO_4_ presence. Carotenoids are synthesized in the chloroplast by the action of a series of nuclear-encoded membrane proteins, and Varela *et al*.^30^ mentioned that some microalgae have the ability to accumulate carotenoids under unfavourable conditions.

**Table 2:** Impact of different magnesium concentrations on cell growth, nutrient removal efficiencies, and pigments.

MgSO_4_ (mM)	Algal biomass^a^ (g L^−1^)	Chlorophyll-a^b^ (mg L^−1^)	Total Carotenoid^c^ (mg L^−1^)	PO_4_-P removal^d^ (%)	NH_3_-N removal^e^ (%)
(Mean ± SD^*^), n = 3					
Control	*1*.*22* ± *0*.*25*	*18*.*5* ± *0*.*9*	*3*.*3* ± *0*.*45*	*75*.*1* ± *5*.*5*	*85*.*7* ± *7*.*6*
0.4	1.36 ± 0.28	**15**.**4** ± **1**.**1**	**7**.**4** ± **0**.**9**	69.2 ± 4.1	88.3 ± 9.1
2	1.02 ± 0.15	**14**.**4** ± **1**.**3**	**6**.**6** ± **0**.**85**	**65**.**2** ± **3**.**6**	85.2 ± 9.6
3.5	1.1 ± 0.20	**12**.**7** ± **1**.**1**	**6**.**1** ± **0**.**8**	66.7 ± 4.9	85.6 ± 10.5
5.1	1.15 ± 0.19	**11**.**1** ± **0**.**8**	**5**.**8** ± **0**.**71**	**55**.**2** ± **3**.**9**	78.1 ± 8.8

^*^SD: Standard Deviation

Bold font indicates statistically significant difference with respect to control MgSO_4_ concentration (t-test, P < 0.05).

The highest nutrient removal for NH_3_-N and PO_4_-P were observed at 0.4 mM MgSO_4_ with 88.3% and 69.2% removal efficiency, respectively. Magnesium is one of the chemicals being studied due to its potential for P and N removal in municipal wastewater treatment. Its reaction mechanism is the same as other chemical precipitation processes^31^. Moreover, Huang *et al*.^32^ reported that Mg^2+^ dominates algal cell absorption and phosphorus utilization. However, in this study, magnesium addition did not improve neither nitrogen nor phosphorus removal compared to control and iron cultivation batches.

**Figure 3:**
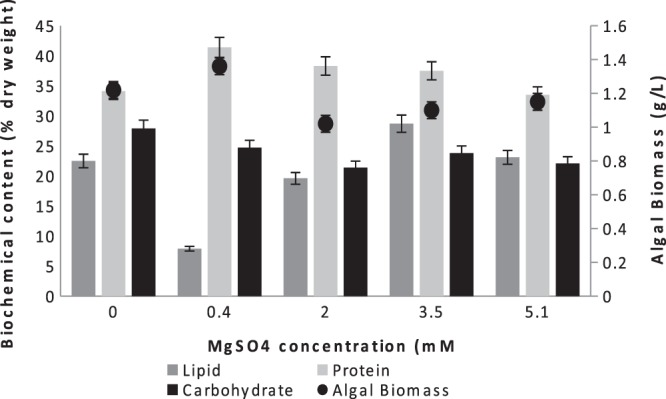
Biochemical composition and biomass amount of the mixed culture at different MgSO_4_ concentrations.

The biochemical components of microalgae can be also influenced by the different concentrations of magnesium in the culture medium. However, in this study there were fluctuations between Mg concentration and biochemical composition. The lipid amount increased by 27% when the magnesium concentration was increased from 0 mM to 3.5 mM; however, it started to decrease at 5.1 mM MgSO_4_ presence. Huang *et al*.^32^ also observed that the supplementation of 100 μM Mg^2+^ to the culture medium increased the lipid content of *Monoraphidium* sp. This result indicated that Mg^2+^ has the potential to stimulate lipid accumulation in microalgae but the threshold of the concentration where it starts to cause inhibition should be determined. Ulloa *et al*.^33^ and Sydney *et al*.^34^ also mentioned that the addition of an appropriate concentration of Mg^2+^ to the culture medium increases the lipid content and lipid productivity of microalgal cells, whereas excess Mg^2+^ decreases the lipid content and lipid productivity (Fig. 3).

The fatty acid compositions mainly consisted of C16:0, C18:0, C18:1 and C18:2; however, Mg^2+^ addition decreased the amount of fatty acids under all concentrations. The highest C16:0 was observed at control with 176.2 ppm, where it was 55.1, 43.2, 42.6 and 27.5 ppm for 0.4, 2, 3.5 and 5.1 mM MgSO_4_ concentrations, respectively (Table 3).

**Table 3:** Fatty acid compositions under different Mg concentrations.

MgSO_4_ concentration (mM)	Fatty acid compositions (ppm) (Mean ± SD*), n = 3			
	C16:0	C18:0	C18:1n9c	C18:2 9c
Control	*176*.*2* ± *12*.*1*	*19*.*09* ± *2*.*5*	*37*.*88* ± *5*.*99*	*55* ± *6*.*23*
0.4	**55**.**1** ± **3**.**5**	**3**.**8** ± **0**.**44**	33 ± 4.35	**22**.**5** ± **3**.**22**
2	**43**.**2** ± **5**.**8**	**2**.**36** ± **0**.**18**	29.64 ± 4.12	**17**.**76** ± **1**.**98**
3.5	**42**.**6** ± **4**.**7**	**0**.**86** ± **0**.**05**	**20**.**1** ± **3**.**11**	**14**.**8** ± **2**.**12**
5.1	**27**.**5** ± **3**.**3**	**0**.**55** ± **0**.**04**	**5**.**6** ± **0**.**76**	**12**.**4** ± **1**.**99**

^*^SD: Standard Deviation

Bold font indicates statistically significant difference with respect to control MgSO_4_ concentration (t-test, P < 0.05).

Addition of 0.4 mM MgSO_4_ to ALD was found to be the most convenient concentration for mixed culture due to higher biomass and higher carotenoid amount compared to the control batch.

### Effect of iron and magnesium on microbial composition

Algal microbiome of mixed culture was measured for the optimum concentration of iron and magnesium, and compared with the control growth. Multi-marker metabarcoding approach was used for microbial community analysis by analyzing four different markers: 16S rRNA, 23S chloroplast rRNA, 18S rRNA and tufA. Photosynthetic microorganisms like cyanobacteria and microalgae have been considered important in the production of valuable co-products along with biofuels in an economically effective and environmentally sustainable way by improving their high value products^35^. In our mixed algae cultures, *Diphylleia rotans*, *Synechocystis PCC-6803* and *Chlorella sorokiniana* were found to be mostly abundant species, as confirmed by 18S rRNA, 16S rRNA, 23S chloroplast rRNA and tufA marker analyses. When iron supplementation was applied on the mixed algal culture, the abundances of the same dominant species were diminished or increased. The abundance of *Synechocystis PCC-6803* and *Chlorella sorokiniana* increased approximately 1.4 folds and 2,5 folds, respectively, for both 16S and 23S rRNA, while *Diphylleia rotans* abundance did not change noticeably. Most abundant microorganisms detected in the mixed cultures were shown in Table 4, and their phylogenetic trees are given in Fig. S2.

**Table 4:** Most abundant microorganisms (>2% abundance) in the cultures based on the taxonomic classification by QIIME 2 analysis.

Marker genes	Genus / species	% abundance		
		Control	Iron Batch	Magnesium Batch
**16 S rRNA**	*Synechocystis PCC-6803*	**20**.**14**	**27**.**51**	**20**.**02**
	*Cyanobium PCC-6307*	**5**.**75**	0.07	0.07
	*Chlorella sorokiniana*	**1**.**79**	**3**.**93**	**2**.**84**
	*Dechloromonas fungiphilus*	**3**.**14**	0.01	0.01
	*Burkholderiaceae*	**2**.**55**	0.84	**3**.**86**
	*Desulfovibrio oxamicus*	**2**.**00**	0.01	0.03
	*Thauera*	0.00	**3**.**28**	0.94
	*Azospirillum*	**5**.**02**	0.01	0.03
	*Burkholderiaceae*	**2**.**55**	0.84	**3**.**86**
	*Thermomonasfusca*	0.85	0.01	**3**.**21**
	*Phycisphaeraceae SM1A02*	0.10	**2**.**39**	0.08
	*Planctomicrobium piriforme*	0.54	**2**.**28**	1.91
	*Turneriella*	0.19	0.89	**4**.**07**
	*Microtrichaceae IMCC26207*	0.09	**3**.**16**	0.31
	*Sediminibacterium*	0.06	0.27	**2**.**30**
	*Gemmatimonas*	**2**.**18**	1.14	1.04
**18 S rRNA**	*Diphylleia rotans*	**45**.**54**	**42**.**17**	**2**.**02**
	*Trebouxiophyceae*	**10**.**14**	**19**.**55**	**12**.**03**
	*Leptophryidae* sp. *WaAra*	**7**.**60**	0.05	0.04
	*Cercozoa* sp. *1 YG-2013*	**6**.**57**	0.18	**7**.**06**
	*Fungi*	**4**.**64**	0.03	0.02
	*Poterioochromonas malhamensis*	**3**.**65**	0.02	0.02
	*Characium saccatum*	**2**.**66**	0.24	**6**.**62**
	*Poteriospumella*	**1**.**92**	0.02	0.00
	*Spongomonas*	1.58	**7**.**52**	1.27
	*Nuclearia*	0.25	**4**.**91**	1.36
	*Amoebozoa* sp. *Pa18*	0.40	0.32	**18**.**17**
	*Chlamydomonas noctigama*	0.12	0.19	**18**.**11**
	*Paraphysoderma sedebokerense*	0.84	0.49	**13**.**22**
**23 S rRNA**	*Synechocystis PCC-6803*	**59**.**69**	**81**.**85**	**80**.**71**
	*Cyanobium PCC-6307*	**28**.**64**	0.32	0.39
	*Chlorella sorokiniana*	**2**.**18**	**5**.**95**	**5**.**93**
	*Planctomicrobium piriforme*	0.33	**5**.**34**	**4**.**71**
**tufA**	*s_Chlorella sorokiniana*	**26**.**72**	**59**.**09**	**31**.**23**
	*c_Chlorophyceae*	**3**.**85**	**32**.**16**	**38**.**06**

The aim of metabarcoding is not only characterizing the communities and biodiversity with high sensitivity, but also detecting the community dynamics, including the interactions between microalgae and other microorganisms. Some studies indicated that presence of bacteria in mixed algal culture can increase algal production and microalgal-bacterial interactions may lead to increased levels of microalgae species and algal production of valuable compounds^36^. Bacteria relationships on algal growth can be mutualistic or parasitic, and knowledge of these mechanisms can be used in order to enhance the algal biomass and high value products^37–39^.

The algae-bacteria microbiome can have key roles for modulating microalgal populations by promoting microalgae growth. In 16 S rRNA analysis, *Synechocystis PCC-6803* was identified in the control sample with highest abundance (20.14%), similar to an abundance of 20% for the same species in the magnesium added culture. This demonstrated that magnesium had no effect on the dominancy *of Synechocystis PCC-6803*. However, with iron addition, the abundance of *Synechocystis PCC-6803* increased up to 27.51%. This increase was reflected on the protein amount of the mixed culture since *Synechocystis PCC-6803* has high protein content (65%)^40^. Another cyanobacteria *Cyanobium PCC-6307* was identified in the control culture with an abundance of 5.75%; however, with iron and magnesium addition, it was not able to survive at all. Moreover, *Chlorella sorokiniana* was present in the control sample with 3.5% abundance and the dominancy increased more than twice, up to 8.46% when iron was added; and up to 5.26% when magnesium was added. This increase was also reflected on the protein amount of the mixed culture since the dry weight analysis of *C*. *sorokiniana* shows that it has 40% protein, 30–38% carbohydrate and 18–22% lipid content^41^. Moreover, bacterial species belonging to *Proteobacteria*, *Spirochaetes*, *Actinobacteria*, *Bacteroidetes* and *Gemmatimonadetes* phyla had different abundance in control, magnesium and iron added conditions. The total abundance of proteobacteria in the control sample was 43% approximately, whereas this abundance decreased to approximately 32% and 40% for the mixed culture when iron and magnesium was added, respectively. In the control culture, the abundance of *Azosprillium* bacteria was 5%, *Dechloromonas fungiphilus* species was 3.14%, *Desulfovibrio oxamicus* specie was 2%, *Burkholderiaceae* bacteria was 2.55% and *Gemmatimonas* bacteria was 2.18%. These most abundant bacteria species found in the mixed culture belong mostly to *Proteobacteria* family, which indicated that algae-proteobacteria interactions were dominated in control environment when there were neither iron nor magnesium addition. In the iron added environment, the abundance of *Proteobacteria* species decreased, leaving *Thauera* species to be present in 3.28%, which was not detected in the control sample. Moreover, *Planctomycetes* phyla of bacteria had been found in iron-added environment together with *Phycisphaeraceae* and *Planctomicrobium* bacteria with 2.39% and 2.28% abundances, respectively. In addition, one *Actinobacteria* phylum bacteria belonging to *Microtrichaceae* family has emerged with 3.16% abundance in the iron added environment. The amount of *Proteobacteria* was higher in the magnesium added environment, where *Thermomonas fusca* species have adapted to the environment and survived with 3.21% abundance. The two *Planctomycetes* bacteria that emerged under iron stress was not detected under magnesium stress with 16S rRNA analysis, however with 23S rRNA analysis, *Planctomicrobium piriforme* bacteria was detected as abundant as in iron stress cultures. In addition, *Turneriella* genus of *Spirochaetes* was found to be abundant in magnesium stress culture with 4.07% and *Sediminibacterium* genus of *Bacteroidetes* was abundant with 2.3%.

In 18S rRNA analysis, heterotrophic flagellate *Diphylleia rotans* was identified as the most dominant algae species in both control sample and iron added sample, with abundances of 45.54% and 42.17% respectively. However, the dominance was diminished in magnesium added culture to an abundance of 2.02%, which clearly demonstrated that magnesium had a negative effect on *Diphylleia rotans*. On the other hand, *Chlamydomonas noctigama* emerged with 18.11% abundance, *Amoebozoa* sp. *Pa18* species emerged with 18.17% and also a fungi species, *Paraphysoderma sedebokerense*, emerged with 13.22% abundance in response to magnesium addition. These results concluded that macro elements such as iron and magnesium could not only affect the concortia of mixed cultures but also wiped out or emerge microorganisms facultatively. *Poterioochromonas malhamensis*, which is known to be a microalgal predator^42^, was detected only in the control culture. *Poterioochromonas malhamensis* can feed on *Chlorella* sp., which was consistent with the low amount of *Chlorella* sp. in the control sample. Therefore, one of the reasons of the increase in the dominance of *Chlorella sorokiniana* species in different environmental stress conditions might be the disappearance of *P*. *malhamensis* species. *Characium saccatum* is another green algae species, which was detected with 2.66% abundance in the control culture and increased up to 6.62% with magnesium addition. However, the abundance of *Characium saccatum* decreased to almost zero with iron addition. Different amoeboid organisms were also detected in 18S rRNA analysis such as *Leptophryidae* sp. *WaAra* were detected with 7.6% dominance in only control sample, while an unclassified *Cercozoa* sp. *1 YG-2013* was detected both in the control culture and magnesium added culture with 6.57% and 7.06% abundances, respectively; which implied a negative effect of iron on *Cercozoa* sp. *1 YG-2013*. Flagellated protozoa *Spongomonas* genus and *Nuclearia* genus were detected with highest abundance in the iron added culture, whereas it was very low at control and magnesium added samples, which indicated a positive effect of iron on *Spongomonas* genus and *Nuclearia* genus.

23S rRNA analysis identified *Synechocystis PCC-6803* as the dominant species for all conditions with the same amount of increase when iron and magnesium were added. However, the second dominant species *Cyanobium PCC-6307* disappeared when iron and magnesium was added as it was also observed in 16S rRNA results which supported the accuracy of results between different markers.

In tufA analysis, a curated database specialized to detect algal species was used^43^ which identified the green algae *Chlorella sorokiniana* as the most dominant algal species with 26.7, 59, and 31.2% relative abundance for control, iron and magnesium batch, respectively.

In conclusion, the effect of iron and magnesium replete on mixed culture can be explained as the acceleration of the growth of protein-rich species, such as *Chlorella sorokiniana*, due to the higher need of protein synthesis, leading to improve high protein content of the total algal biomass. Moreover, iron and magnesium addition did not increase neither lipid nor carbohydrate and they were both the highest at control batch (Figs. 2 and 3). According to 23 S rRNA results, At control batch *Cyanobium PCC-6307* relative abundance was 28.64%, where it was 0.32% and 0.39% when iron and magnesium were added, respectively. This big difference on relative abundance of *Cyanobium PCC-6307* in different growth media clarified the reason of higher amount of lipid and carbohydrate observed at control batch comparing to other batches.

### Principal component analysis

Principal component analysis (PCA), which is useful for discerning patterns within the species viability data itself, was applied to observe the relation between the measured parameters and the dominant species in different growth conditions.

**Figure 4:**
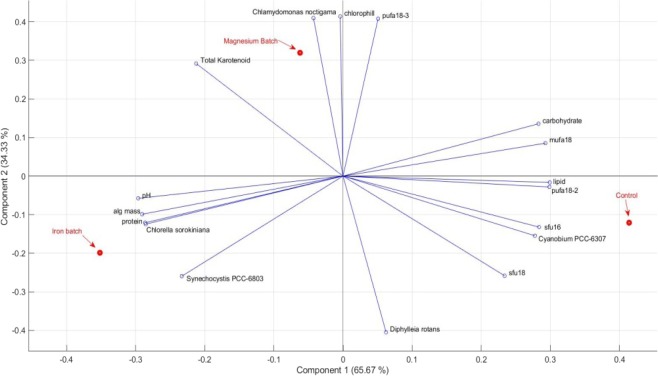
PCA biplot for various parameters measured for the mixed culture with different growth media conditions.

The PCA components for each culturing condition were plotted in relation to the biochemical composition, pigment composition and relative abundances of microbial species detected in highest amount via each of 16S, 18S and tufA marker analysis. Mostly abundant cyanobacteria Synechocystis PCC-6803 and Cyanobium PCC-6307, heterotrophic flagellate Diphylleia rotans, green microalgae Chlamydomonas noctigama and Chlorella sorokiniana were included in the PCA biplot (Fig. 4).

Iron effect on the mixed culture was shown to be correlated with the protein content as well as with the abundances of *Chlorella sorokiniana* and *Synechocystis PCC-6803*, which are both protein-rich species. Magnesium effect, on the other hand, was shown to be mostly correlated with the pigments as well as with the abundance of *Chlamydomonas noctigama*. The control batch was correlated with the lipid content as well as with the abundance of *Cyanobium PCC-6307*. The fatty acid (FA) compositions of the mixed cultures were dependent on the taxonomic abundances and cultivation conditions. PCA identified the abundance of *Cyanobium PCC-6307* as the major source of variability within the control culture. This variability could be mostly explained by the differences in SFU (C16, C18), MUFA (C18:1), and PUFA (C18:2) contents. C18:3 content was shown to be increased in magnesium batch, which was also observed as positively correlated with the magnesium batch sample in the PCA biplot (Fig. 3). Poerschmann *et al*.^44^ also reported that polyunsaturated fatty acids, especially linolenic acid (18:3), was the most abundant in their study with *Chlamydomonas* sp.

## Conclusion

Successful cultivation of microalgae on wastewater, particularly on digestate, requires close monitoring since each wastewater has its own characteristics. In this study, the microbial community profiles and dynamics were first identified via metabarcoding of 16S rRNA, 18 S rRNA, 23S chloroplast rRNA an tufA regions. The community profiles changed drastically due to the macro element addition where the differences in the mixed algal community can be helpful for the adaptation to different environmental and growth conditions. 3.1 mM FeSO_4_ and 0.4 mM MgSO_4_ addition was found to be the optimum concentration with a positive effect on growth and biochemical composition. Moreover, the dynamics of undefined algal microbiome grown in anaerobic digestate showed significant changes and demonstrated how symbiotic life can be changed if macro elements were added to the ALD. Therefore, this algal microbiome might be a solution for both reducing adverse effect of anaerobic liquid digestate and lowering the cost of microalgae production to help commercialisation of algae-derived products.

## Materials and Methods

### Wastewater collection and analysis

The liquid digestate was obtained from a full scale plant decomposing the waste mixture of mechanically/manually separated organic fraction of municipal solid waste (50%), cattle manure (17%), leaching water from solid waste collection vehicles (8%), expired market wastes (4%) and chicken manure (4%). The characterization of ALD was given in a previous work by Ermis and Altinbas^5^. The PerkinElmer Optima 7000 DV ICP (Inductively Coupled Plasma) optical emission spectrometer was used to analyze the wastewater elements using the standard solutions where calcium (Ca) was 58.1 mg L^−1^, magnesium (Mg) was 19.1 mg L^−1^, iron (Fe) was 27.8 mg L^−1^, manganese (Mn) was 3.9 mg L^−1^, aluminum (Al) was 9.5 mg L^−1^, silicon (Si) was 46.7 mg L^−1^, lead (Pb) was 1.1 mg L^−1^, boron (B) was 5.8 mg L^−1^, chromium (Cr) was 5.3 mg L^−1^, cadmium (Cd) was 0.4 mg L^−1^, nickel (Ni) was 3.1 mg L^−1^, silver (Ag) was 4.8 mg L^−1^, sulfur (S) was 619.7 mg L^−1^, zinc (Zn) was 0.9 mg L^−1^, Sr was 1 mg L^−1^ and sodium (Na) was 871.4 mg L^−1^.

### Isolation and identification of mixed microalgae

The isolation of mixed culture of microalgae was performed as described in a previous study^5^. Algal cultures were firstly inoculated in M_8_ media containing the following components (per liter): KNO_3_ (3000 mg L^−1^), KH_2_PO_4_ (740 mg L^−1^), Na_2_HPO_4_.2H_2_0 (260 mg L^−1^), CaCl_2_.2H_2_O (13 mg L^−1^), Fe EDTA (10 mg L^−1^), FeSO_4_.7H_2_O (130 mg L^−1^), MgSO_4_.7H_2_O (400 mg L^−1^), and 1 mL Micronutrients consisting of Al_2_(SO_4_)_3_.18H_2_O (3.58 g L^−1^), MnCl_2_.4H_2_0 (12.98 g L^−1^), CuSO_4_.5H_2_O (1.83 g L^−1^), and ZnSO_4_.7H_2_O (3.2 g L^−1^)^8^. Afterwards, mixed culture was inoculated in diluted ALD (2%) for acclimation. The culture was inoculated into the same diluted wastewater repeatedly and monitored by microscopic observations frequently. Before the beginning of the each batch cultivation, it was assured that the culture was healthy.

Isolated wild-type microalgae culture was firstly checked by light microscopy, and mixed culture was morphologically characterized by using microalgae systematics books. Afterwards, next generation sequencing was performed.

### PCR amplification and sequence analyses of 16 S rRNA, 18S rRNA, 23S rRNA and tufA

Molecular confirmation of isolates was performed via next generation sequencing of 16S/18S/23S rRNA and tufA marker regions. Genomic DNA from different mixed microalgae culture samples was isolated and high-throughput sequencing analysis was applied to each sample. Targeted amplicon libraries were constructed with universal V4 region primers [515 f (F), 5’-GTGCCAGCMGCCGCGGTAA-3’ and 806r (R), 5’-GGACTACHVHHHTWTCTAAT-3’] for 16S, TAReuk454FWD1 (F), 5’-CCAGCASCYGCGGTAATTC-3’ and TAReukREV3 (R), 5’-ACTTTCGTTCTTGATYRA-3’ primers for 18S rDNA, p23SrV_f1 (F), 5’-GGACAGAAAGACCCTATGAA-3’ and p23SrV_r1 (R), 5’-TCAGCCTGT-TATCCCTAGAG-3’ primers for 23S rDNA, (F) 5’-TGAAACAGAAMAWCGTCATT-3’ and (R) 5’-CCTTCNCGAATMGCRAAW-3’ primers for elongation factor tufA. Purified-amplicon libraries were sequenced using an Illumina MiSeq platform (2×300 paired-end reads).

### Bioinformatic analysis

Sequence data from marker regions were analysed with Quantitative Insights Into Microbial Ecology 2 program (QIIME2 ver. 2019.4^45^). After demultiplexing raw reads with cutadapt plug-in, denoising and generation of amplicon sequence variants (ASVs) were performed using the Divisive Amplicon Denoising Algorithm (DADA2). Denoising step includes chimera detection and removal, sequence error elimination, singleton exclusion and sequence trimming based on sequence quality graph and expected amplicon size. The resulting sequences were then classified with the SILVA reference database^46,47^ (132_release of Dec 13, 2017) and tufA database^43^. For 16 S rDNA analysis, SILVA database trimmed to the V4 region (515F/806R) was used for taxonomic classification. Taxonomic bar plots are given in Fig. S1.

After taxonomic classification, alpha and beta diversity analysis among samples were performed via “qiime diversity core-metrics-phylogenetic” function. To allow for a comparison between the analysis of different samples, we used a user-specified sampling depth per sample per marker analysis. The sample sequences were rarefied (sub-sampled) to 45000, 48000, 50000 and 7000 reads for 16S, 18S, 23S and tufA analyses respectively (Table S1).

Microalgae consortia in this study was identified via amplicon sequencing of small subunit of eukaryotic nuclear ribosomal DNA (18S rDNA), small subunit of prokaryotic ribosomal DNA and eukaryotic chloroplast DNA (16 S rDNA), large subunit of eukaryotic chloroplast DNA (23S rDNA), and elongation factor EF-Ttu (tufA) gene of prokaryotic (cyanobacteria) and eukaryotic algae.

Alpha diversity of microbes, including phylogenetic diversity was tested to document whether the internal diversity differs among different environmental stress conditions, including iron and magnesium stress. For this purpose, several alpha diversity parameters were tested. Alpha diversity indices (observed ASV richness, Shannon diversity, Faith’s phylogenetic diversity, and Pielou’s evenness) of rarefied samples were calculated in QIIME2 with q2-diversity plug-in. Next, maximum-likelihood phylogenetic trees were constructed with “align-to-tree-mafft-fasttree” function of phylogeny plug-in, which uses FastTree2 Next, maximum-likelihood phylogenetic trees were constructed upon masked MAFFT alignment of representative sequences, and rooted phylogenies were inferred via “fasttree” and “midpoint-root” functions of phylogeny plug-in, which uses FastTree2^48^. Phylogenetic trees, constructed using sequences with most abundant ASVs (minimum total feature frequency of 100), were uploaded to the Interactive Tree of Life (iTOL) tool^49^ for the illustration of the taxonomic community compositions (Fig. S2).

Beta diversity among the three samples was analyzed as well in order to test statistically whether microbial composition differed among the three conditions. Beta diversity metrics were calculated in QIIME 2 with q2-diversity plug-in and beta function. Principal coordinates analysis (PCoA) was employed based on Bray-curtis distance matrix in order to detect the variation in the microbial communities of samples (Fig. S3).

Principal component analysis (PCA) was also performed in MATLAB R2015a. The PCA plot was constructed in order to demonstrate the differential effects of culturing conditions on the mostly detected microbial species identified in the mixed culture. The percent compositions of lipid, protein, carbohydrate and pigments (carotenoid and chlorophyll) under magnesium and iron added cultures, along with the percent relative abundances of detected microbial species were standardized by z-score normalization prior to PCA analysis.

### Experimental design

The iron and magnesium source and the starting point concentrations were determined based on the synthetic media study by authors (data not shown). Ferrous sulfate (FeSO_4_.7H_2_O, Molar Mass: 278 g/mol) starting amount was based on the amount available in M_8_ media (130 mg L^−1^) and gradually increased to 1000 mg L^−1^ of FeSO_4_; and magnesium sulfate (MgSO_4_.7H_2_O, Molar Mass: 246.5 g/mol) starting amount was based on the amount contained in BG-11media (130 mg L^−1^) and gradually increased to 1000 mg L^−1^ of MgSO_4_^50^. The mixed algal culture was operated in batch culture with triplicate using 1000 ml Erlenmeyer flask with 800 ml working volume with different doses of FeSO_4_and MgSO_4_ concentrations. The molarity calculations showed that the FeSO_4_ concentrations were 0.6, 1.8, 3.1, and 4.5 mM (130, 400, 700, and 1000 mg L^−1^ FeSO_4_); and the MgSO_4_ concentrations were 0.4, 2, 3.5 and 5.1 mM (75, 400, 700 and 1000 mg L^−1^ MgSO_4_). The best growth dilution ratio was selected as 10% for mixed culture in a previous study by authors (data not shown) and according to the ICP results, the iron amount was negligible for 10% ALD; hence, 10% ALD was assumed as control batch (0 mM concentration) for both iron and magnesium batch.

All experiments were started with 2.5 mg chl-a L^−1^ and 0.5 ± 0.1 g L^−1^ algal biomass and harvested at the end of the stationary phase after 16 days by centrifuging at 5000 rpm for 10 minutes. Cultures were kept in an acclimation cabinet under approximately 150 µmol photon m^−2^ s^−1^ continuous illumination measured with a light meter (Hansatech QRT1 Quantitherm), at 25 °C ± 2 °C during the acclimation period and during the experiments^5^, where continuous light was provided to increase algal growth. All batches were monitored for more days to observe the death phase to confirm that the stationary phase ended.

### Analytical methods

Nitrogen was measured as Total Kjeldahl Nitrogen (TKN) and Ammonia (NH_3_-N); whereas Phosphorus was measured as Total Phosphorus (TP) and Orthophosphate (PO4). Suspended Solid, TKN, NH_3_-N, TP and PO_4_-P values were analyzed as mg L^−1^ according to Standard Methods^51^.

Total protein content was estimated by the method of Lowry^52^ where samples were pre-boiled for 10 minutes with 2 N NaOH in 1:1 ratio (v/v) as a pretreatment. 1 ml of cooled sample was taken and 700 μl of Lowry solution was added. After vortexing, samples were kept in the dark for 20 minutes at room temperature. Folin solution was prepared 5 minutes before the end of 20 minutes, and 100 µl of folin solution was added to the mixture and vortexed. The samples were left in the dark for at least 30 minutes more and read at spectrometer (750 nm) against the distilled water.

Total carbohydrate was determined by Anthrone reagent method^53^ where 1 ml of the sample was mixed with 2 ml of 75% sulfuric acid and 4 ml of anthrone solution, and incubated for 15 minutes at 100 °C. After samples were cooled down, they were read against distilled water at 630 nm by spectrometer.

Total lipids were calculated by a slightly modified version of Bligh and Dyer’s method^54^. Wet biomass containing 100 ± 5 mg was taken and 1.25 ml of chloroform and 2.5 ml of methanol were added. After 20 minutes of shaking, the samples were vortexed with 1.25 mL of chloroform. The samples were vortexed again by adding 1.25 ml of distilled water. After centrifuging the samples at 3000 rpm for 10 minutes, the lower phase was removed and the samples were evaporated at 70 ° C in a vacuum oven. The glass containing lipid was kept 1 hour at 105 °C to reach constant weight. Triplicate samples were analyzed and the average values were taken.

Pigment contents were determined via centrifugation of 2 ml of microalgae cells of each strain at 5000 rpm for 5 min. Pellet was taken and suspended with 2 ml methanol (90%). The mixture was incubated in water bath at 80 °C for 5 min. The steps were continued by centrifugation at 10000 rpm for 5 min. The supernatant was transferred and measured by UV-Vis spectrophotometer at 470 nm, 665 nm and 655 nm against the solvent (methanol) blank. The concentration of chlorophyll a (chl-a), chlorophyll b and total carotenoids (car) were calculated as explained by Sumanta *et al*.^55^.

For Fatty Acid Methyl Ester (FAME) analyses, Laurens *et al*.^56^ and El-Shimi *et al*.^57^ procedures were followed for acid catalyzed *in-situ* transesterification with 5–10 mg of microalgal biomass. Methyl tridecanoate (C13: 0ME) was prepared as an internal standard and 20 μL C13:0 ME (10 mg/mL), 200 μL chloroform: methanol (2: 1, v/v), and 300 μL 0.6 M HCl:methanol (methanolic hydrochloric acid) were added on 5–10 mg dry algal samples and incubated for 1 hour at preheated 85 °C heat block. At the end of the incubation, 1 ml of hexane was added and the upper phase was measured by Gas Chromotography (GC). Shimadzu AOC-20i, GC 2010 model Gas Chromatography with CN100 capillary column (Teknokroma, Barcelona, Spain) with a length of 100 m × 0.25 mm and an internal diameter of 0.2 μm film thickness were used. The carrier gas was helium and the hydrogen gas flow was 40 ml / min whereas air gas flow was 400 ml/min.

## Supplementary information

Supplementary Information.
