# Author Correction: Effect of iron and magnesium addition on population dynamics and high value product of microalgae grown in anaerobic liquid digestate

**DOI:** 10.1038/s41598-020-69043-6

**Published:** 2020-07-15

**Authors:** Hande Ermis, Unzile Guven-Gulhan, Tunahan Cakir, Mahmut Altinbas

**Affiliations:** 1 Department of Environmental Engineering, Istanbul Technical University, 34469 Maslak, Istanbul, Turkey; 2 PHI Tech Bioinformatics R&D Inc., 41400 Gebze, Kocaeli Turkey; 3 Department of Bioengineering, Gebze Technical University, 41400 Gebze, Kocaeli Turkey

Correction to: *Scientific Reports* https://doi.org/10.1038/s41598-020-60622-1, published online 26 February 2020

The Article contains several typographical errors and omitted References in the Materials and Methods section under the subheading ‘PCR amplification and sequence analyses of 16 S rRNA, 18S rRNA, 23S rRNA and tufA’ where:

“PCR amplification and sequence analyses of 16 S rRNA, 18S rRNA, 23S rRNA and tufa

Molecular confirmation of isolates was performed via next generation sequencing of 16S/18S/23S rRNA and tufA marker regions. Genomic DNA from different mixed microalgae culture samples was isolated and high-throughput sequencing analysis was applied to each sample. Targeted amplicon libraries were constructed with universal V4 region primers [515 f (F), 5′-GTGCCAGCMGCCGCGGTAA-3′ and 806r (R), 5′-GGACTACHVHHHTWTCTAAT-3′] for 16S, TAReuk454FWD1 (F), 5′-CCAGCASCYGCGGTAATTC-3′ and TAReukREV3 (R), 5′-ACTTTCGTTCTTGATYRA-3′ primers for 18S rDNA, p23SrV_f1 (F), 5′-GGACAGAAAGACCCTATGAA-3′ and p23SrV_r1 (R), 5′-TCAGCCTGT-TATCCCTAGAG-3′ primers for 23S rDNA, (F) 5′-TGAAACAGAAMAWCGTCATT-3′ and (R) 5′-CCTTCNCGAATMGCRAAW-3′ primers for elongation factor tufA. Purified-amplicon libraries were sequenced using an Illumina MiSeq platform (2 × 300 paired-end reads).”

should read:

“PCR amplification and sequence analyses of 16S rRNA, 18S rRNA, 23S rRNA and tufa

Molecular confirmation of isolates was performed via next generation sequencing of 16S/18S/23S rRNA and tufA marker regions. Genomic DNA from different mixed microalgae culture samples was isolated and high-throughput sequencing analysis was applied to each sample. Targeted amplicon libraries were constructed with universal V4 region primers [515f (F), 5′-GTGYCAGCMGCCGCGGTAA-3′^1^ and 806r (R), 5′-GGACTACNVGGGTWTCTAAT-3′^2^] for 16S, TAReuk454FWD1 (F), 5′-CCAGCASCYGCGGTAATTC-3′ and TAReukREV3 (R), 5′-ACTTTCGTTCTTGATYRA-3′ primers^3^ for 18S rDNA, p23SrV_f1 (F), 5′-GGACAGAAAGACCCTATGAA-3′ and p23SrV_r1 (R), 5′-TCAGCCTGTTATCCCTAGAG-3′ primers^4^ for 23S rDNA, (F) 5′-TGAAACAGAAMAWCGTCATT-3′ and (R) 5′-CCTTCNCGAATMGCRAAW-3′ primers for elongation factor tufA. Purified-amplicon libraries were sequenced using an Illumina MiSeq platform (2 × 300 paired-end reads).”

The omitted References are listed below as References 1–4 respectively.
