# Supplementary material for: Effect of iron and magnesium addition on population dynamics and high value product of microalgae grown in anaerobic liquid digestate
Source: Sci Rep. 2020 Feb 26;10:3510. doi: 10.1038/s41598-020-60622-1 (PMC7044283; doi:10.1038/s41598-020-60622-1)
Supplement: Supplementary file 1 — Supplementary Information. [file 41598_2020_60622_MOESM1_ESM.docx]

**Supplementary Information**

**Effect of iron and magnesium addition on population dynamics and high value product of microalgae grown in anaerobic liquid digestate**

Hande Ermis^1^*, Unzile Guven-Gulhan^2^, Tunahan Cakir^3^, Mahmut Altinbas^1^

^1^ Departmant of Environmental Engineering, Istanbul Technical University, 34469, Maslak, Istanbul, Turkey.

^2^ PHI Tech Bioinformatics R&D Inc., 41400, Gebze, Kocaeli, Turkey.

^3^ Department of Bioengineering, Gebze Technical University, 41400, Gebze, Kocaeli, Turkey

*Corresponding Author e-mail: hande_ermis@hotmail.com, phone:+905355286064

**
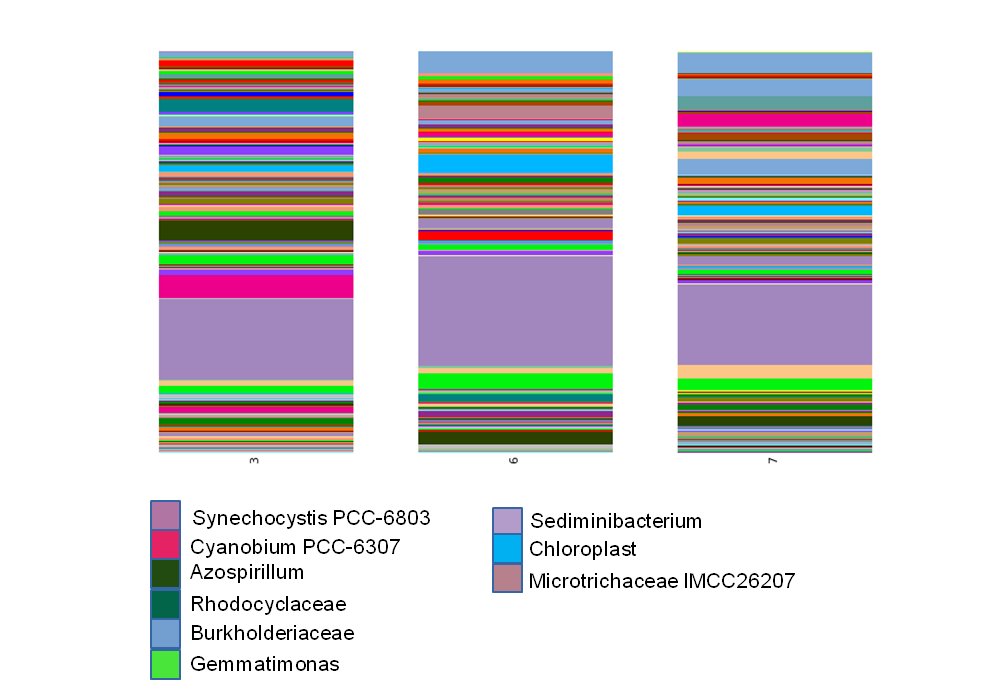
**

**(a)**

**
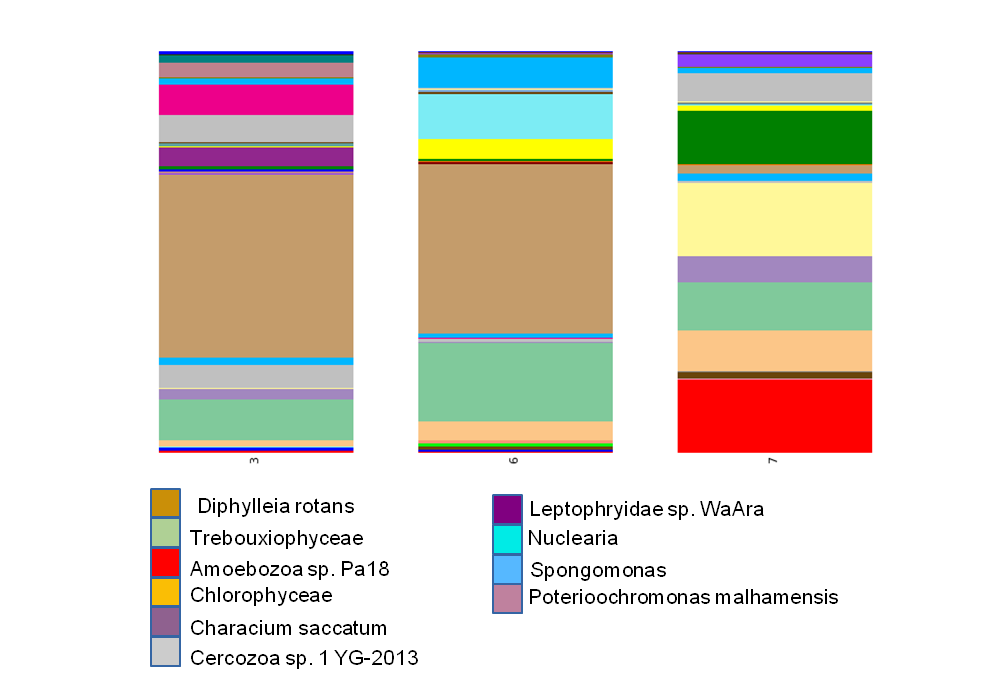
**

**(b)**

**
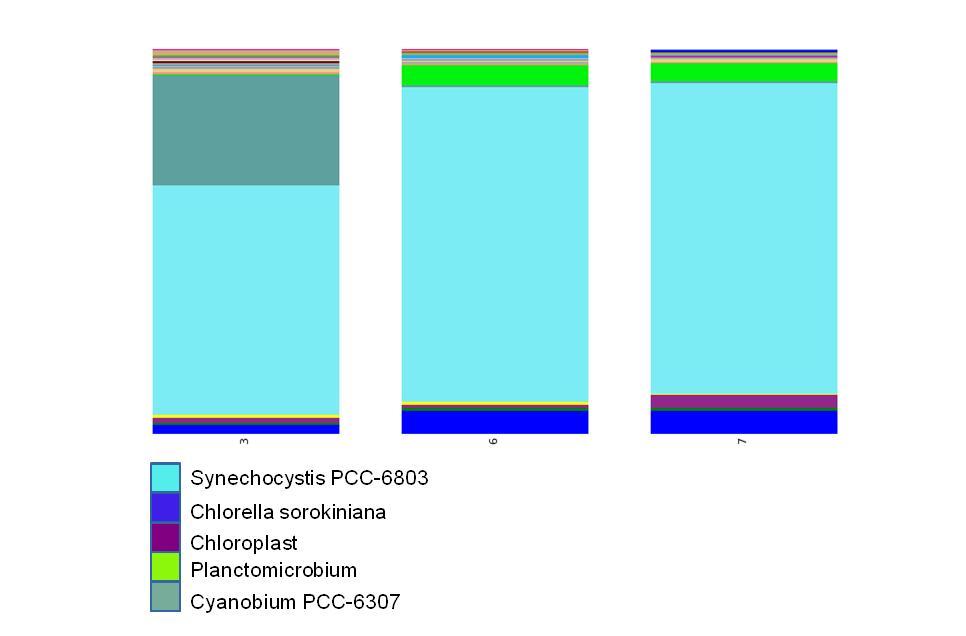
**

**(c)**

**
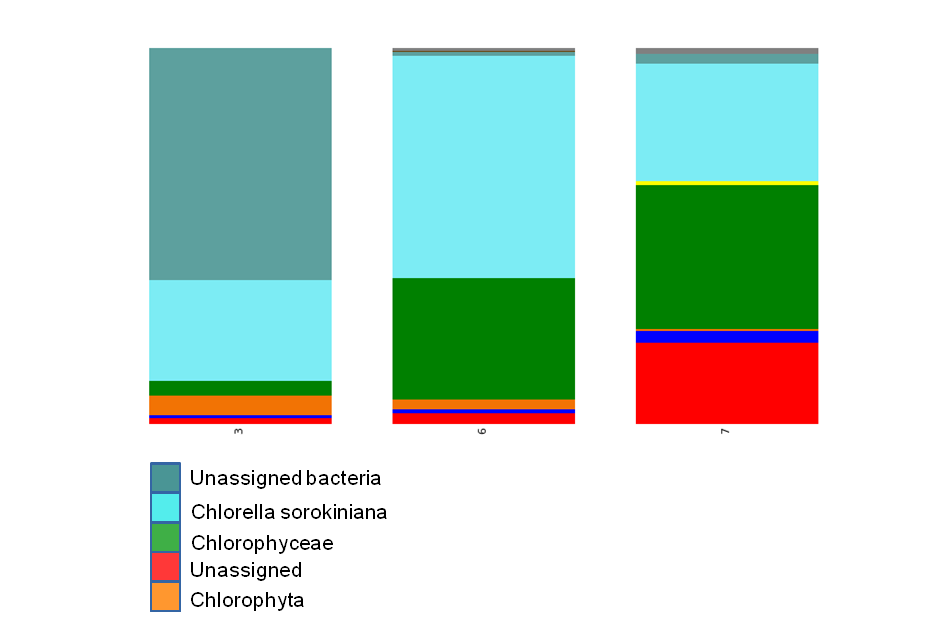
**

**(d)**

**Figure S1.** Bar plots showing variation in the relative abundances of bacterial taxonomy in mixed microalgae microbial communities (3, 6 and 7). Colors represent microbial taxonomy classified by Silva taxonomy (release_132) with using **(a)** 16S rDNA marker regions, **(b)** 18S rDNA marker regions and **(c)** 23S rDNA marker regions, by **(d)** tufA database [32]

Note: 2% and above abundant species/order are shown in the legend.

**
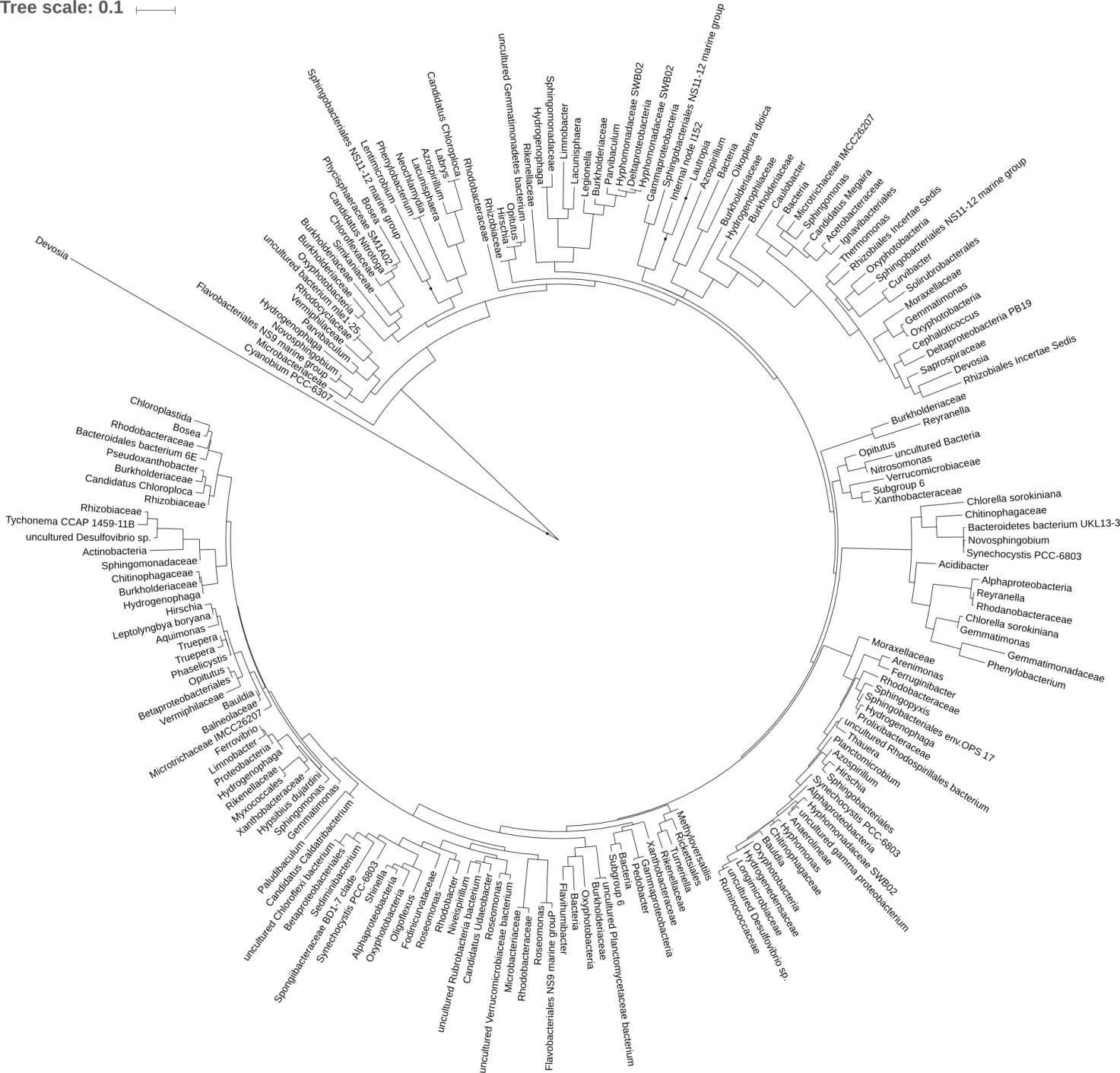
**

**(a)**

**
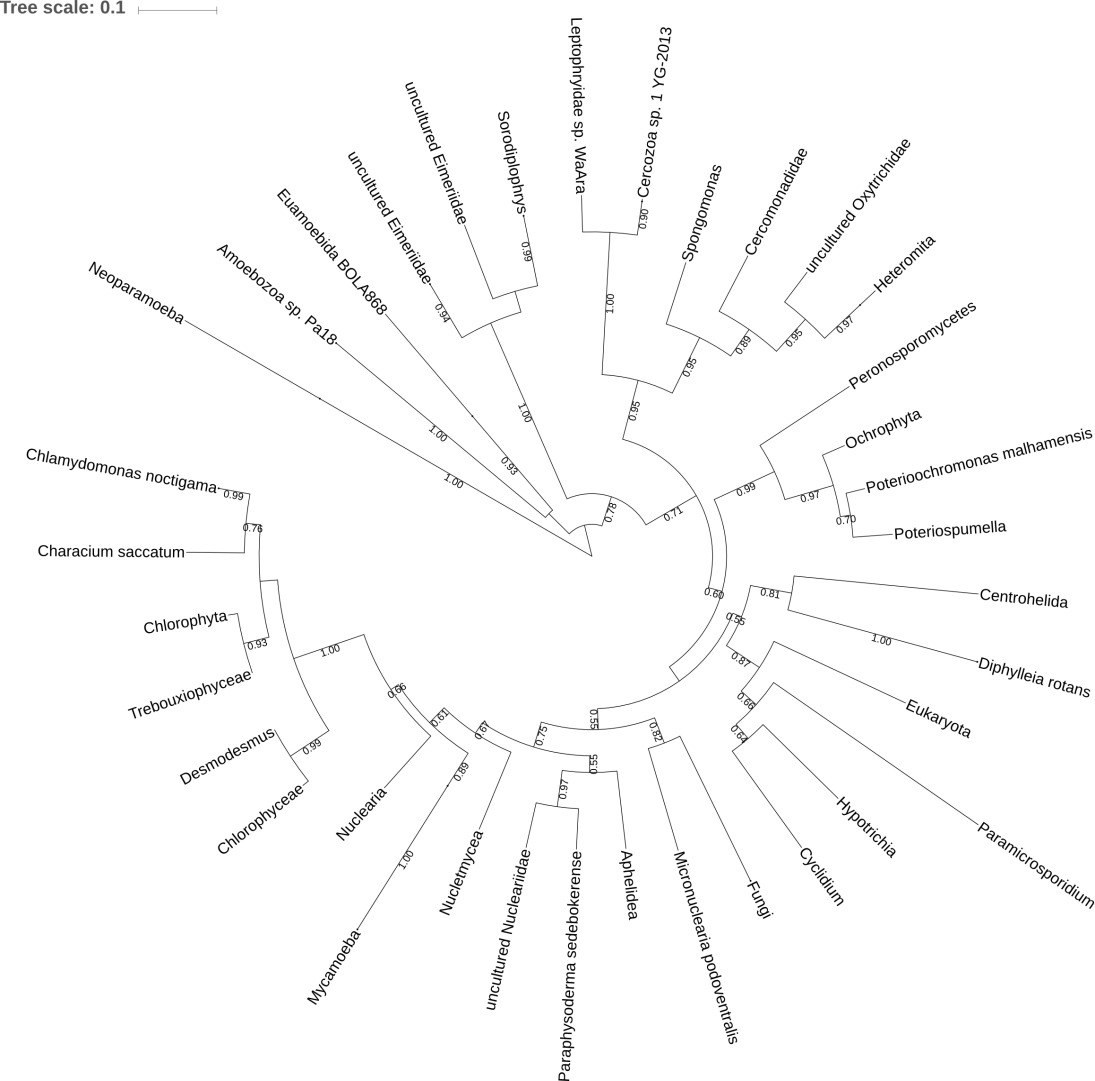
**

**(b)**

**
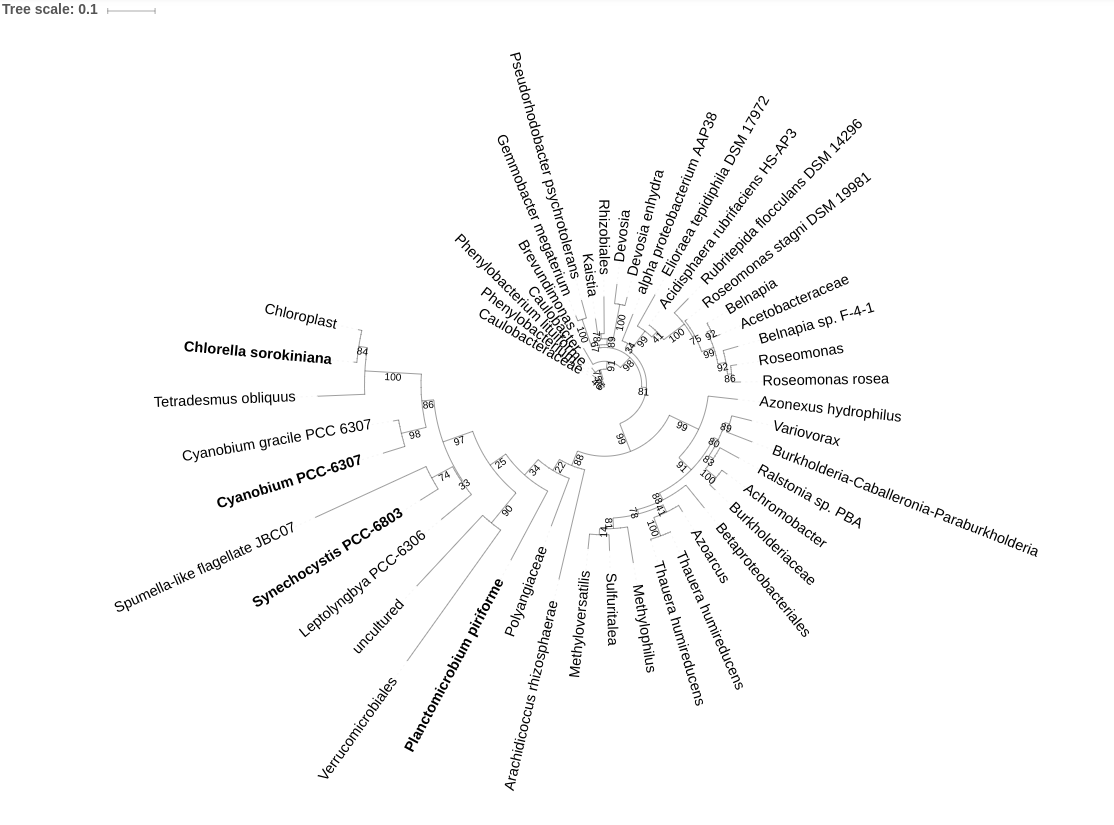
**

**(c)**

**
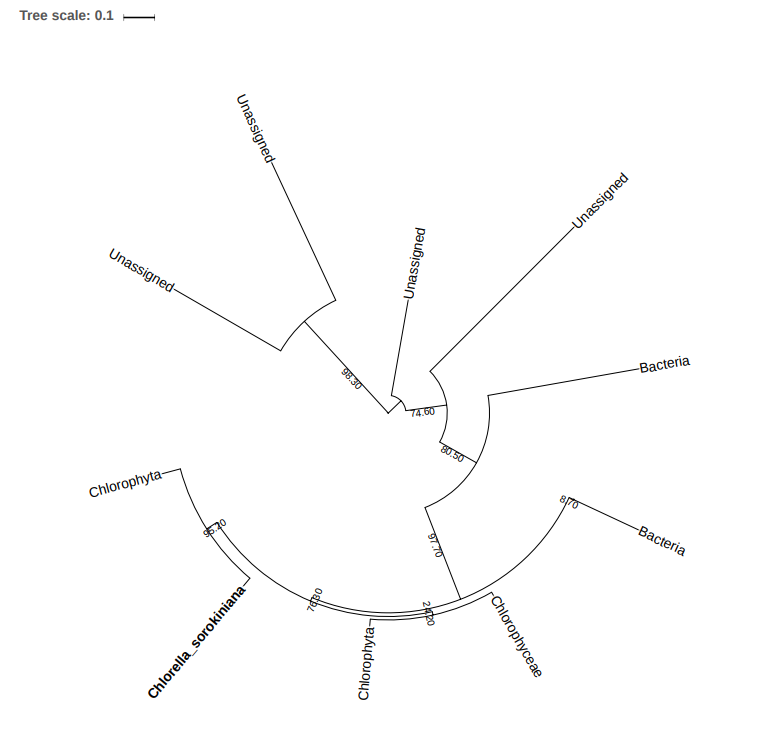
**

**(d)**

**Figure S2.** Phylogenetic trees showing the relationship of (**a)** 16S rRNA, **(b)** 18S RNA, **(c)** 23S rRNA and **(d)** tufA gene sequences. All the phylogenetic trees except for 23S rRNA were constructed with a minimum total feature frequency of 100. Phylogenetic analyses were performed with maximum-likelihood method and visualized using iTOL.

**Note:** Most frequent 23S rRNA taxonomic classifications involved mostly unassigned bacteria, thus no filtering criteria was applied. On the other hand, most of the taxonomic classifications of tufA gene sequences were found to be unassigned, thus most frequent features were shown in the figure.

**
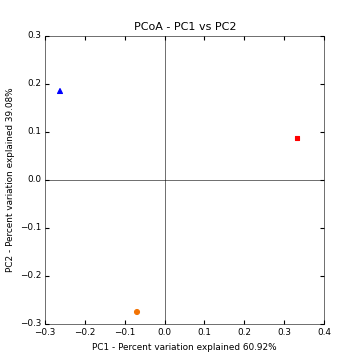

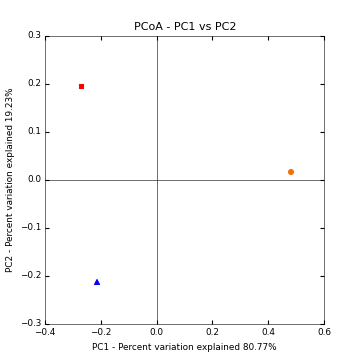
**

Magnesium batch

Iron batch

Control

**(a)**

**(b)**

**(c)**

**
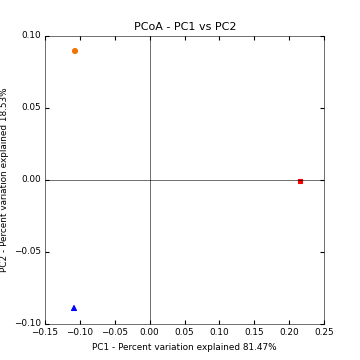

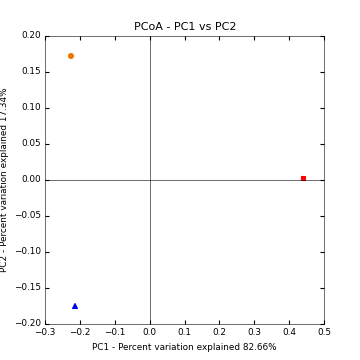
**

**(d)**

**Figure S3.**  Principal-coordinate analysis (PCoA) performed for **(a)** 16S rRNA, **(b)** 18S rRNA, **(c)** 23S rRNA and **(d)** tufA marker regions based on Bray-curtis distance matrix.

**Table S1.** Sequence information from QIIME 2 processing of NGS amplicon reads

|  | Samples | Number of reads | Number of sequences after denoising with Dada2 | Number of observed ASVs |
| --- | --- | --- | --- | --- |
| 16S rRNA | 3 | 59133 | 52716 | 663 |
|  | 6 | 51853 | 46515 | 511 |
|  | 7 | 64518 | 53712 | 603 |
| 18S rRNA | 3 | 70261 | 48786 | 83 |
|  | 6 | 100345 | 62326 | 71 |
|  | 7 | 151331 | 98323 | 79 |
| 23S rRNA | 3 | 74095 | 52190 | 87 |
|  | 6 | 102271 | 80584 | 77 |
|  | 7 | 93255 | 71565 | 74 |
| tufA | 3 | 14610 | 11014 | 45 |
|  | 6 | 12500 | 8016 | 40 |
|  | 7 | 15564 | 7623 | 91 |
